# Comparison of (Partial) economic evaluations of transforaminal lumbar interbody fusion (TLIF) versus Posterior lumbar interbody fusion (PLIF) in adults with lumbar spondylolisthesis: A systematic review

**DOI:** 10.1371/journal.pone.0245963

**Published:** 2021-02-11

**Authors:** Inge J. M. H. Caelers, Suzanne L. de Kunder, Kim Rijkers, Wouter L. W. van Hemert, Rob A. de Bie, Silvia M. A. A. Evers, Henk van Santbrink

**Affiliations:** 1 CAPHRI School for Public Health and Primary Care, Maastricht University, Maastricht, The Netherlands; 2 Department of Neurosurgery, Zuyderland Medical Centre, Sittard-Geleen, Heerlen, The Netherlands; 3 Department of Neurosurgery, Maastricht University Medical Centre, Maastricht, The Netherlands; 4 Department of Primary and Community Care, Centre for Family Medicine, Geriatric Care and Public Health, Radboud University Medical Centre, Nijmegen, The Netherlands; 5 Department of Orthopedic Surgery, Zuyderland Medical Centre, Sittard-Geleen, Heerlen, The Netherlands; 6 Department of Epidemiology, Maastricht University, Maastricht, The Netherlands; 7 Department of Health Services Research, CAPHRI Care and Public Health Research Institute, Maastricht University, Maastricht, The Netherlands; 8 Centre for Economic Evaluation, Trimbos Institute, Netherlands Institute of Mental Health and Addiction, Utrecht, The Netherlands; Universidad Nacional Autonoma de Nicaragua Leon, NICARAGUA

## Abstract

**Introduction:**

The demand for spinal fusion surgery has increased over the last decades. Health care providers should take costs and cost-effectiveness of these surgeries into account. Open transforaminal lumbar interbody fusion (TLIF) and posterior lumbar interbody fusion (PLIF) are two widely used techniques for spinal fusion. Earlier research revealed that TLIF is associated with less blood loss, shorter surgical time and sometimes shorter length of hospital stay, while effectiveness of both techniques on back and/or leg pain are equal. Therefore, TLIF could result in lower costs and be more cost-effective than PLIF. This is the first systematic review comparing direct and indirect (partial) economic evaluations of TLIF with PLIF in adults with lumbar spondylolisthesis. Furthermore, methodological quality of included studies was assessed.

**Methods:**

Searches were conducted in eight databases for reporting on eligibility criteria; TLIF or PLIF, lumbar spondylolisthesis or lumbar instability, and cost. Costs were converted to United States Dollars with reference year 2020. Study quality was assessed using the bias assessment tool of the Cochrane Handbook for Systematic Reviews of Interventions, the Level of Evidence guidelines of the Oxford Centre for Evidence-based Medicine and the Consensus Health Economic Criteria (CHEC) list.

**Results:**

Of a total of 693 studies, 16 studies were included. Comparison of TLIF and PLIF could only be made indirectly, since no study compared TLIF and PLIF directly. There was a large heterogeneity in health care and societal perspective costs due to different in-, and exclusion criteria, baseline characteristics and the use of costs or charges in calculations. Health care perspective costs, calculated with hospital costs, ranged from $15,867-$43,217 in TLIF-studies and $32,662 in one PLIF-study. Calculated with hospital charges, it ranged from $8,964-$51,469 in TLIF-studies and $21,838-$93,609 in two PLIF-studies. Societal perspective costs and cost-effectiveness, only mentioned in TLIF-studies, ranged from $5,702/QALY-$48,538/QALY and $50,092/QALY-$90,977/QALY, respectively. Overall quality of studies was low.

**Conclusions:**

This systematic review shows that TLIF and PLIF are expensive techniques. Moreover, firm conclusions about the preferable technique, based on (partial) economic evaluations, cannot be drawn due to limited studies and heterogeneity. Randomized prospective trials and full economical evaluations with direct TLIF and PLIF comparison are needed to obtain high levels of evidence. Furthermore, development of guidelines to perform adequate economic evaluations, specified for the field of interest, will be useful to minimize heterogeneity and maximize transferability of results.

**Trial registration:**

**Prospero-database registration number:** CRD42020196869.

## Introduction

With the growing possibilities in health care, the question arises whether increasing costs, due to new expensive treatment options, are justified given limited financial resources. In this regard, spinal fusion surgery is a field in which new expensive techniques are emerging quickly. Spinal fusion surgery can be conducted in case of (expected) instability of the lumbar spine, which can result in slippage of vertebrae (lumbar spondylolisthesis). This might lead to nerve entrapment, resulting in neurological complaints. Lumbar instability and spondylolisthesis with nerve entrapment are the most common indications to perform instrumented spinal fusion surgery [1, 2]. During this surgery, compression of the nerves will be relieved, and vertebrae are stabilized by placing screws and rods.

Grotle et al. described an increased rate of spinal fusion surgery of 154% in the Norwegian population from 1999 to 2013 [3]. The incidence of spondylolisthesis increases with age due to spinal degeneration, which implies that the demand for spinal fusion surgery will rise further in the near future due to the aging of the population [4]. The higher demand of spinal fusion surgery leads to increased medical costs; previous studies concerning the national US bill for spinal fusion surgery have shown a 7.9 fold increase between 1998 and 2008 and a 2.8 fold increase between 2004 and 2015 [5, 6]. Therefore, physicians should use the best, but also the most cost-effective methods, to keep health care affordable.

Two widely used techniques for spinal fusion surgery are transforaminal lumbar interbody fusion (TLIF) and posterior lumbar interbody fusion (PLIF). These open (non-minimal invasive) procedures differ in approach of the intervertebral space to remove the intervertebral disc; the TLIF procedure makes use of a unilateral route to the intervertebral space, instead of a bilateral approach for PLIF [7–9].

Previous literature, including our recently published systematic review, showed equal effectiveness on back and/or leg pain of TLIF and PLIF, resulting in the same improvement in quality of life [10–19]. Furthermore, TLIF is associated with less blood loss, shorter surgical time and possible shorter length of hospital stay (LOS) compared to PLIF, which could result in lower medical costs [10–19]. Therefore, TLIF could be more cost-effective than PLIF, when considering the lower costs and equal improvement in quality of life. To our knowledge, there is currently no systematic review available comparing direct and indirect (partial) economic evaluations of open-TLIF and PLIF. Expanding our knowledge of cost-effectiveness, along with clinical effectiveness of TLIF and PLIF, can assist to choose the superior method in future daily practice.

The aim of this systematic review is to compare (partial) economic evaluations of TLIF and PLIF, directly and indirectly, in adults with lumbar spondylolisthesis. Furthermore, the methodological quality of the included studies will be assessed.

## Methods

### Review protocol

This systematic review was executed in accordance with the PRISMA statement and the five-step approach on preparing a systematic review of (partial) economic evaluations by Van Mastrigt et al. [20–24]. Before the start of the study, the protocol was discussed with co-authors and has been published in the PROSPERO-database (registration number CRD42020196869). The protocol consisted of a research question, search strategy and eligibility criteria for assessing full-text studies and held the following research questions:

1. *Does transforaminal lumbar interbody fusion (TLIF) in adults with lumbar spondylolisthesis result in lower costs and/or is it more cost-effective than posterior lumbar interbody fusion (PLIF) when comparing direct and indirect (partial) economic evaluations*?
2. *What is the methodological quality of the included studies*?

### Search strategy and eligibility criteria

In collaboration with a medical information specialist (GF; see Acknowledgements), a comprehensive systematic literature search of the following databases was performed: Medline (using Pubmed), Embase (using Ovid), Cochrane Library, Current Controlled Trials, ClinicalTrials.gov, NHS Centre for Review and Dissemination, Econlit and Web of Science. Detailed search strategies are available in S1 File. Our last search was conducted on July 6^st^, 2020. Partial and full economic evaluations were included if they described all of the following eligibility criteria: (i) TLIF (transforaminal lumbar interbody fusion) or PLIF (posterior lumbar interbody fusion), (ii) lumbar spondylolisthesis or lumbar instability, (iii) cost. Full economic evaluations compare two or more interventions and describe both costs and effects. In partial economic evaluations, costs and/or effects are considered, but they do not involve a comparison between interventions and do not relate costs to benefits [20]. Furthermore, studies describing TLIF or PLIF versus another intervention were also included, while both direct and indirect comparisons of (partial) economic evaluations were eligible. No restrictions were set on language, publication status or time period of the literature search. Only full text studies were included in the final analysis.

### Study selection and data collection process

Selection of studies was performed by two authors (IC and SdK). First, duplicate studies were removed. Second, potential studies were screened on title and abstract. Third, for final inclusion, full text screening on all eligibility criteria was performed.

Data were collected using a prospectively designed data collection sheet, independently extracted by two authors (IC and SdK). To determine costs and cost-effectiveness, the following data items were considered: country of origin, study design, study population, follow-up time, utility measurement tool, gained Quality Adjusted Life Years (QALYs), cost sources, index year for cost conversion, currency, health care and societal perspective costs, total costs and costs per QALY (cost-effectiveness). Possible discounting and sensitivity analysis of the included studies were rated in the Consensus Health Economic Criteria (CHEC) list. If necessary, consensus was reached between both authors through discussion.

Cost-effectiveness was determined as the total costs of a treatment divided by the difference in obtained health benefit (QALY-gain). Total costs can be determined by combining health care perspective costs (costs for health care resources that an intervention requires, like physician time) with societal perspective costs (all resource costs associated with an intervention, including costs for caregiver time or absenteeism) [25]. Cost-effectiveness of two interventions can be compared using the incremental cost-effectiveness ratio (ICER). In this ratio, difference in costs between two interventions will be divided by their difference in effect [26]. All costs were converted to United States Dollars with reference year 2020, based on the IMF World Economic Outlook Database, with the use of a web-based tool developed by the Campbell and Cochrane Economics Methods Group (CCEMG) and the Evidence for Policy and Practice Information and Coordinating Centre (EPPI-Centre) (v.1.6) [27]. If the index year was not mentioned in the study, the last year of patient inclusion was used for this calculation. If last year of patient inclusion was not described, the year of publication was used as index year.

### Quality assessment

Two authors (IC and SdK) evaluated the selected studies. Risk of bias was assessed with the bias assessment tool of the Cochrane Handbook for Systematic Reviews of Interventions [28]. Studies were scored as “low”, “high” or “unclear” risk of bias, based on six different domains. Level of evidence was determined with the guidelines of the Oxford Centre for Evidence-based Medicine (2011) [29]. The methodological quality of the (partial) economical evaluations was analyzed using the Consensus Health Economic Criteria (CHEC) list [30]. The CHEC-list consists of 19 categories, including discounting and sensitivity analysis, where a single point can be assigned to each category with a maximum score of 19 points. A CHEC-list score between 15 and 17 points is rated as average methodological quality [30]. If necessary, consensus was reached between both authors through discussion.

### Statistical analysis

Above mentioned data items of each included study were independently collected by two authors using a prospectively designed data collection sheet. Reported results of costs, QALY-gain and cost-effectiveness were presented in ranges.

## Results

### Overview of studies

#### Study selection

**Fig 1:**
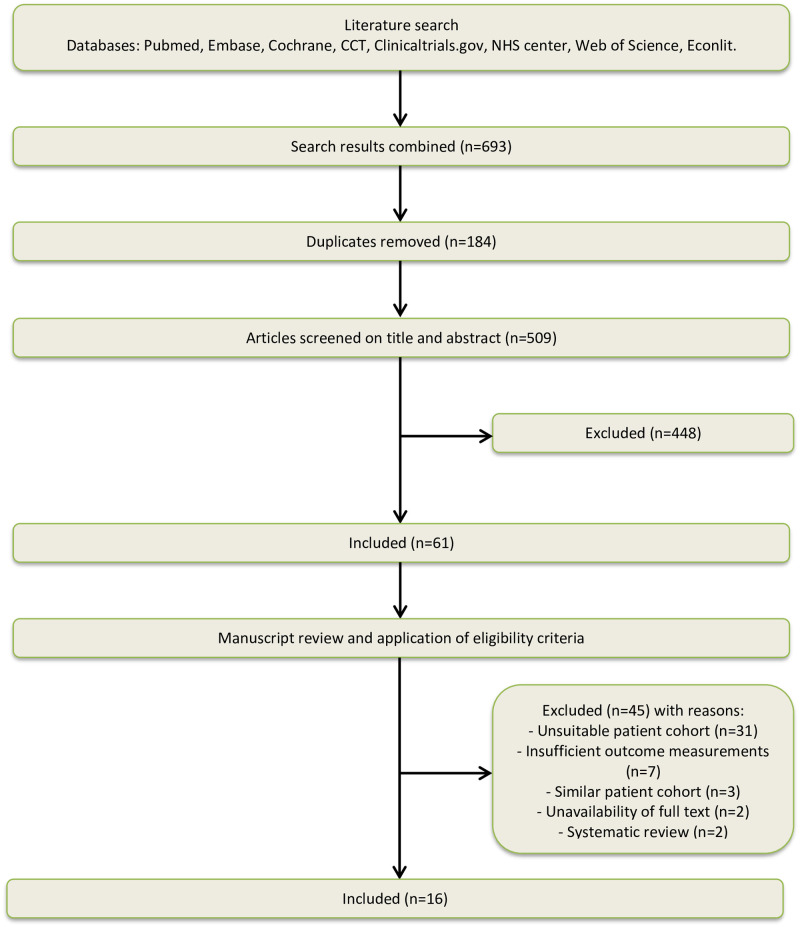
Flowchart of study selection.

Results of the study selection process are summarized in Fig 1. Database searching resulted in identification of 693 studies. Sixty-one studies were eligible for full text screening. After full text analysis, 45 studies were excluded; for 31 studies it was unclear if they included patients with lumbar spondylolisthesis, for seven studies the outcome measurements did not include costs, for three studies the patient cohorts were similar, for two studies no full text was available and two studies were systematic reviews. Sixteen studies were univocally included for final analysis.

#### Study characteristics

Study characteristics of the 16 included studies are summarized in S1 Table. Nine studies had a cost-effectiveness design [31–39] and seven were cost analyses [40–46]. Publication years ranged from 2001 to 2020. Follow-up time ranged from time of hospitalization to two years postoperative. Fifteen studies were performed in the United States of America using United States Dollars [31, 33–46] and one was performed in Europe (Denmark) using Euros [32]. There were no (partial) economic evaluations that compared TLIF and PLIF directly. For this reason, only indirect comparison of TLIF and PLIF was possible. Thirteen studies compared TLIF with other instrumented spine surgery techniques [31–39, 42–45]. Three studies compared PLIF with other techniques [40, 41, 46]. Comparators are described in S2 Table and excluded from further analysis, while this is beyond the scope of this systematic review.

Results of the included studies are summarized in S2 Table. Health care perspective cost sources included hospital financial departments, Medicare (official site U.S. government), Diagnosis Related Groups codes (DRG codes), Current Procedural Terminology codes (CPT codes), national health insurance service registrations and Redbook (health care drug pricing resource). The majority of studies used hospital costs (actual hospital expenditures for treatment) to determine health care perspective costs [32, 34, 36–39, 41, 43, 45]. Seven studies (five TLIF-studies and two PLIF-studies) used hospital charges (amount charged by the hospital for treatment) or a mixture of costs and charges to calculate health care perspective costs [31, 33, 35, 40, 42, 44, 46].

Societal perspective costs and total costs were mentioned in six TLIF-studies only [31–33, 36–38]. To determine societal perspective costs, different calculations were used; five studies used the Human Capital Approach, in which every hour not worked counts as an hour lost [31, 33, 36–38, 47], while one study used the DREAM database, which bases information of absenteeism on public transfer payments administered by Danish ministries. Loss of productivity was determined by length of paying sick benefit [32, 48]. Finally, one study included loss of workdays, loss of housekeeping days and unpaid caregiver opportunity costs in their calculation [37]. Five studies included one or two of these factors [31–33, 36, 38].

Nine TLIF-studies reported mean or cumulative QALY gain measured with EQ-5D, SF-6D or ODI (Oswestry Disability Index) [31–39].

Cost-effectiveness was calculated in three TLIF-studies [31, 33, 38]. Since none of the studies compared TLIF and PLIF directly, there was no ICER available.

### Quality of identified studies

Risk of bias and methodological quality of (partial) economical evaluations (CHEC-list) are summarized in S2 File. Fifteen studies had a high risk of bias [31, 33–46]. The study of Christensen et al., the only randomized controlled trial, had low risk of bias. Furthermore, this was the only study reaching evidence level 2 [32]. All other studies reached evidence level 3 or 4.

When analyzed with the CHEC-list, the mean quality score of the studies was 10.5 (5.5–15.0) out of 19 points. Christensen et al. was the only study of average quality with a score between 15 and 17 points [32]. Three out of 16 studies included discounting in their analysis [32, 35, 41]. Sensitivity analysis was performed in two studies [32, 33].

#### Study results

Results of studies are summarized in S2 Table. Results of the randomized controlled trial of Christensen et al. on TLIF are reported separately [32]. Health care perspective costs were calculated in all included studies, ranging from $8,964 to $93,609. Based on hospital costs, it ranged from $15,867 to $43,217 in TLIF-studies and $32,662 in one PLIF-study [34, 36–38, 41, 43]. Based on hospital charges, it ranged from $8,964 to $51,469 in TLIF-studies and $21,838 to $93,609 in two PLIF-studies [31, 33, 35, 40, 42, 44]. Calculations of Christensen et al., based on hospital costs, resulted in $34,436 for health care perspective costs [32].

Societal perspective costs for TLIF ranged from $5,702 to $21,478 [31, 33, 36–38]. Christensen et al. described societal perspective costs of $48,538 [32].

Total costs (health care and societal perspective costs combined) for TLIF ranged from $39,120 to $52,941 [31, 33, 36–38]. Christensen et al. described a total of $82,973 [32].

Cumulative two-year QALY gain for TLIF ranged from 0.14 QALY to 0.86 QALY [31, 33, 34, 37, 38].

Cost-effectiveness for TLIF ranged from $50,092/QALY to $90,977/QALY [31, 33, 38]. Societal perspective costs, total costs, QALY gain and cost-effectiveness were not reported for PLIF.

## Discussion

To our knowledge, this is the first systematic review that compares (partial) economic evaluations of open-TLIF and PLIF in adults with lumbar spondylolisthesis and assesses the methodological quality of the included studies. After a broad search, no (partial) economic evalutions were found that compared TLIF and PLIF directly. For this reason, only indirect comparison was possible. Overall risk of bias was high and methodological quality was low, except for the trial of Christensen et al. [32]. Therefore, results of Christensen et al. were separately described. Due to heterogeneity in health care and societal perspective costs of the other eligible studies, results were described in ranges. Costs described by Christensen et al. and the other included studies differed most in societal perspective costs ($48,538 versus $5,702-$21,478) [31–33, 36–38]. The most important reason for this difference was the length of absenteeism, which was the longest in the study of Christensen et al. (34.2 weeks). In this study, absenteeism was based on data of the DREAM database instead of the Human Capital Approach, which resulted in higher total costs [32]. Cost-effectiveness was only mentioned in TLIF articles and ranged from $50,092/QALY to $90,977/QALY.

Comparison of studies and transferability of their results was difficult due to overall low quality and large heterogeneity for various reasons [49, 50]. First, hospital costs or hospital charges were used to determine healthcare perspective costs, with hospital costs defined as actual hospital expenditures for treatment and hospital charges defined as the amount charged by hospital for treatment. This is an important and relevant difference; recent publications suggest some US hospitals charge 10 times the costs of services, resulting in an extreme mark-up [51, 52]. Using hospital charges leads to an unrealistic comparison [53]. Second, despite compiled guidelines for economic evaluations of several countries recommending to perform cost-effectiveness studies from the societal perspective, in only six out of the 16 included studies in this review societal perspective costs were calculated. For example, in the United States, the Panel on Cost-Effectiveness in Health and Medicin incorporates both direct (for instance health care costs) and indirect costs (for instance productivity losses), regarding its importance to health insurance, employers and patients [54]. Guidelines regarding cost-effectiveness analyses specified for the field of spinal surgery are needed to minimize heterogeneity and maximize transferability of study results. Third, definitions and sources of costs differed between studies, resulting in a broad cost range. For instance, Christensen et al. calculated healthcare related costs using costs of index hospitalization, costs of diagnostic tests, visits to outpatient clinics and the emergency room up to two years postoperative, resulting in a total of $34,436 [32]. Djurasovic et al. only included costs of index hospitalization, resulting in a total of $15,867 [39]. Finally, differences could also be explained by year of data collection and quality of the studies. The newest studies resulted in the lowest health care related costs for both TLIF and PLIF [35, 39, 45, 46]. The two oldest studies reported the highest healthcare related costs [40, 44]. Furthermore, both studies were evidence level 4 and had the lowest CHEC-list score (5.0 and 7.0). On the other hand, studies with evidence level 3, reported comparable healthcare related costs, ranging from $29,516-$36.468 [31, 33, 36–38]. However, Sulaiman et al., an evidence level 3 study, reported higher costs ($43,217), but had a lower CHEC-list score (10.0) compared to the other level 3 studies (CHEC-list score ranging from 11.5–14.5) [43]. For this reason, we believe that year of data collection and quality of studies can influence results in addition to above mentioned factors.

Comparison of the differences in QALY gain was also diffcult as studies used different questionnaires to calculate QALY, for example EQ-5D, SF-6D or ODI.

Ideally, we would have preferred to include prospective (partial) economic evaluations of TLIF and PLIF in adults with lumbar spondylolisthesis. Unfortunately, an explorative search resulted in a limited number of studies on TLIF and PLIF in a prospective design. We therefore left the prospective study design criterion out of the search to include studies based on any design. Furthermore, ‘costs’ was included as eligibility criterion instead of ‘cost-effectiveness’ to include both partial and full economic evaluations that include costs. Eligible studies had to include either TLIF or PLIF to make indirect comparison possible.

Comparitors of TLIF and PLIF of the included studies are mentioned in S2 Table. Comparing all other instrumented spine surgery techniques with TLIF and PLIF, is beyond the scope of this review. However, the choice of a comparator can bias the outcome of (partial) economic evaluations, since a possible inappropriate comparator may result in a potentially misleading conclusion that the other intervention represents as dominant [55].

This study included only full text, published studies and not conference proceedings, PhD dissertations or other grey literature. This might have resulted in underreporting of available studies. During the full text analysis and data extraction, multiple articles were found from the same research group. However, after thorough review of these studies by two authors (IC and SdK), no reasons were found to assume that the same patients were included in the studies [36–39, 45]. For this reason, all studies were included.

Cost-effectiveness was calculated only for TLIF with a range from $50,092/QALY to $90,977/QALY [31, 33, 38]. The accepted threshold for cost-effectiveness is currently subject of debate and differs per country. However, when using a cost-effectiveness threshold of $50.000/QALY, Gandhoke et al. and Kim et al. reported cost-effectiveness above this threshold and Adogwa et al. around this threshold [31, 33, 38]. Bearing in mind that none of these three TLIF-studies were randomized controlled trials of high quality, firm conclusions cannot be drawn when using the cost-effectiveness threshold. Future recommendations can be made to solve this issue. First, high quality research on cost-effectiveness of TLIF and PLIF is needed to come to a stronger conclusion on whether these surgical methods actually exceed the threshold. Second, there are two suggestions to improve the cost-effectiveness of an intervention when, after thorough and high quality investigation, it shows to be above the threshold. This could be by either reducing costs or improving QALY-gain. The first can be established by gaining insight in cost factors per surgery to determine possibilities for cost reduction. The latter can be reached by optimizing surgical indications to patients. To establish this, clinical guidelines and research on prediction models to predict postoperative clinical outcome, would be useful.

## Conclusion

This is the first systematic review that compares (partial) economic analysis of TLIF and PLIF in adults with lumbar spondylolisthesis. This review shows that both TLIF and PLIF are expensive procedures. Considering the increasing financial burden, rising demand of instrumented spinal fusion surgery and comparable clinical effectiveness of the two techniques, a possible difference in cost-effectiveness would be of great importance. Although TLIF and PLIF are both frequently used techniques for lumbar spondylolisthesis, no (partial) economic evaluations are available directly comparing both techniques. Therefore, it is not possible to discern which technique is less costly and more cost-effective. Randomized prospective trials and economical evaluations directly comparing TLIF and PLIF to obtain high levels of evidence are needed. Quality of life reports with validated instruments and economical evaluations conducted according to standardized approach should enable physicians and decision makers to objectify the clinical results and fill knowledge gaps in this specific area. Furthermore, economic evaluations need to be given more attention within health care education to gain knowledge about this subject.

## Supporting information

S1 Table Study characteristics of included studies. (DOCX)

S2 Table Results of included studies. (DOCX)

S1 File Search results. (DOCX)

S2 File Risk of bias and methodological quality of economical evaluations (CHEC-list). (DOCX)

S1 Checklist PRISMA 2009 checklist. (DOCX)

## Abbrevations

CCEMG: Campbell and Cochrane Economics Methods Group
CHEC: Consensus Health Economic Criteria
CPT codes: Current Procedural Terminology codes
DRG codes: Diagnosis Related Groups codes
EPPI-Centre: Evidence for Policy and Practice Information and Coordinating Centre
EQ-5D: EuroQol 5 Dimensions
ICER: incremental cost-effectiveness ratio
ODI: Oswestry Disability Index
PLIF: posterior lumbar interbody fusion
QALY: Quality Adjusted Life Year
SF-6D: Short Form 6 Dimensions
TLIF: transforaminal lumbar interbody fusion
