# Supplementary material for: Comparison of (Partial) economic evaluations of transforaminal lumbar interbody fusion (TLIF) versus Posterior lumbar interbody fusion (PLIF) in adults with lumbar spondylolisthesis: A systematic review
Source: PLoS One. 2021 Feb 11;16(2):e0245963. doi: 10.1371/journal.pone.0245963 (PMC7877595; doi:10.1371/journal.pone.0245963)
Supplement: S1 Table — (DOCX) [file pone.0245963.s001.docx]

S1 Table. Study characteristics of included studies.

| **Author** | **Country of origin** | **Design Economic evaluation** | **Date collection** | **Follow-up time** | **Number of patients** | | | **Other comparator** | **Mean age (in year)** | **Indication for surgery** | **Level of surgery** | **Level of evidence** | **CHEC-list score** |
| --- | --- | --- | --- | --- | --- | --- | --- | --- | --- | --- | --- | --- | --- |
|  |  |  |  |  |  | ***TLIF*** | ***PLIF*** |  |  |  |  |  |  |
| Whitecloud et al. (2001) | USA | Cost analysis | Retrospective | 1 year | 80 | 40 | - | ALIF + iPLF | 44.7 | Spondylolisthesis, degenerative disc disease, failed back syndrome | 1-level | 4 | 5.5 |
| Wang et al. (2010) | USA | Cost analysis | Retrospective | Time of hospitalization | 59 | - | 15 | MIS-TLIF | 58.0 | Spondylolisthesis, degenerative disc disease, spinal stenosis | 1-level | 4 | 7.0 |
| Adogwa et al. (2011) | USA | Cost-effectiveness study | Prospective | 2 years | 45 | 45 | - | - | 51.0 | Spondylolisthesis | 1-level | 3 | 11.5 |
| Lucio et al. (2012) | USA | Cost analysis | Retrospective | 45 days | 210 | - | 101 | MIS-XLIF | 58.0 | Spondylolisthesis, degenerative disc disease, spinal stenosis, etc. | 2- level | 4 | 7.0 |
| Parker et al. (2012) | USA | Cost-effectiveness study | Prospective | 2 years | 30 | 15 | - | MIS-TLIF | 49.7 | Spondylolisthesis | 1-level | 3 | 14.5 |
| Sulaiman et al. (2014) | USA | Cost analysis | Prospective | 2 years | 68 | 11 | - | MIS-TLIF | 56.4 | Spondylolisthesis | All levels | 3 | 10.0 |
| Singh et al. (2014) | USA | Cost analysis | Retrospective | 60 days | 66 | 33 | - | MIS-TLIF | 49.9 | Spondylolisthesis, degenerative disc disease, spinal stenosis | 1-level | 4 | 11.5 |
| Parker et al. (2014) | USA | Cost-effectiveness study | Prospective | 2 years | 100 | 50 | - | MIS-TLIF | 52.6 | Spondylolisthesis | 1-level | 3 | 14.5 |
| Christensen et al. (2014) | Denmark | Cost-effectiveness study | Prospective | 2 years | 100 | 51 | - | iPLF | 51.0 | Long lasting back-pain (including spondylolisthesis) | - | 2 | 15.0 |
| Gandhoke et al. (2015) | USA | Cost-effectiveness study | Prospective | 2 years | 74 | 45 | - | LLIF | - | Spondylolisthesis, failed back syndrome, etc. | 1-level | 3 | 13.5 |
| Kim et al. (2017) | USA | Cost-effectiveness study | Prospective | 2 years | 99 | 62 | - | PLF | 58.7 | Spondylolisthesis | 1-level | 3 | 12.0 |
| Jazini et al. (2018) | USA | Cost-effectiveness study | Retrospective | 2 years | 62 | 31 | - | ALIF + iPLF | 54.8 | Spondylolisthesis | 1-level | 4 | 10.5 |
| Tye et al. (2018) | USA | Cost-effectiveness study | Retrospective | 1 year | 66 | 25 | - | ALIF + iPLF | 52.4 | Spondylolisthesis | 1-level | 4 | 9.5 |
| Lyons et al. (2019) | USA | Cost analysis | Retrospective | Time of hospitalization | 233 | - | 48 | nPLF/iPLF | 46.2 | Spondylolisthesis, spinal stenosis | 1-level | 4 | 9.0 |
| Djurasovic et al. (2020) | USA | Cost-effectiveness study | Retrospective | 1 year | 66 | 33 | - | MIDLIF | 57.0 | Spondylolisthesis, spinal stenosis, adjacent segment disease, degenerative disc disease | 1-, and 2-level | 4 | 9.5 |
| Ver et al. (2020) | USA | Cost analysis | Retrospective | Time of hospitalization | 156 | 52 | - | MIDLIF/RA-MIDLIF | 53.1 | Spondylolisthesis, spinal stenosis, degenerative disc disease | 1-, and 2-level | 4 | 7.0 |

Abbreviations: TLIF = transforaminal lumbar interbody fusion, PLIF = posterior lumbar interbody fusion, CHEC-list = Consensus Health Economic Criteria-list, ALIF = anterior lumbar interbody fusion, iPLF = instrumented posterolateral fusion, MIS-TLIF = minimally invasive surgery – transforaminal lumbar interbody fusion, MIS-XLIF = minimally invasive surgery - extreme lateral interbody fusion, LLIF = lateral lumbar interbody fusion, nPLF = non-instrumented posterolateral fusion, MIDLIF = midline transforaminal lumbar interbody fusion with cortical screws, RA-MIDLIF = Robot-assisted midline transforaminal lumbar interbody fusion with cortical screws.
