# Supplementary material for: Comparison of (Partial) economic evaluations of transforaminal lumbar interbody fusion (TLIF) versus Posterior lumbar interbody fusion (PLIF) in adults with lumbar spondylolisthesis: A systematic review
Source: PLoS One. 2021 Feb 11;16(2):e0245963. doi: 10.1371/journal.pone.0245963 (PMC7877595; doi:10.1371/journal.pone.0245963)
Supplement: S2 Table — (DOCX) [file pone.0245963.s002.docx]

S2 Table. Results of included studies.

| **Author** | **Utility measurement tool** | **Cost resources** | | **Charges or costs** | **Currency** | **Used Index year for cost converting** | **QALY gain** | **Healthcare perspective costs (US Dollars)** | **societal perspective costs (US Dollars)** | **Total costs (US Dollars)** | **Cost-utility score (US Dollars/QALY)** | **QALY gain** | **Healthcare perspective costs (US Dollars)** | **societal perspective costs (US Dollars)** | **Total costs (US Dollars)** | **Cost-Utility score (US Dollars/QALY)** |
| --- | --- | --- | --- | --- | --- | --- | --- | --- | --- | --- | --- | --- | --- | --- | --- | --- |
|  |  | ***Healthcare perspective*** | ***Societal perspective*** |  |  |  | ***TLIF*** |  |  |  |  | ***PLIF*** |  |  |  |  |
| Whitecloud et al. (2001) | - | Hospital financial department | - | Charges | USD | 1998 | - | 51,469 | - | - | - | - | - | - | - | - |
| Wang et al. (2010) | - | Hospital  financial department | - | Charges | USD | 2010 | - | - | - | - | - | - | 93,609 | - | - | - |
| Adogwa et al. (2011) | EQ-5D | Medicare, DRG codes | Follow up of missed workdays and caregivers | Costs | USD | 2011 | Mean gain 0.43, 2 years cumulative gain 0.86 | 29,516 | 13,541 | 44,058 | 50,092 | - | - | - | - | - |
| Lucio et al. (2012) | - | Hospital financial department | - | Costs | USD | 2009 | - | - | - | - | - | - | 32,662 | - | - | - |
| Parker et al. (2012) | EQ-5D | Medicare, DRG codes, Redbook | Follow up of missed workdays and homemaking days | Costs | USD | 2012 | Mean gain 0.41 | 30,143 | 21,155 | 51,298 | - | - | - | - | - | - |
| Sulaiman et al. (2014) | - | Hospital financial department | - | Costs | USD | 2012 | - | 43,217 | - | - | - | - | - | - | - | - |
| Singh et al. (2014) | - | Hospital financial department | - | Costs and charges | USD | 2010 | - | 28,103 | - | - | - | - | - | - | - | - |
| Parker et al. (2014) | EQ-5D | Hospital financial department, Medicare, DRG codes, Redbook, CPT codes | Follow up of missed workdays, homemaking days and caregivers | Costs | USD | 2014 | Mean gain 0.44, 2 years cumulative gain 0.70 | 31,463 | 21,478 | 52,941 | - | - | - | - | - | - |
| Christensen et al. (2014) | SF-6D | National health insurance service register, DRG codes | DREAM database | Costs | Euro | 2012 | - | 34,436 | 48,538 | 82,973 | - | - | - | - | - | - |
| Gandhoke et al. (2015) | EQ-5D | Hospital financial department | Follow up of missed workdays | Charges | USD | 2013 | Mean gain 0.34, 2 years cumulative gain 0.67 | 36,684 | 14,089 | 49,670 | 73,465 | - | - | - | - | - |
| Kim et al. (2017) | EQ-5D | Medicare, DRG codes, CPT codes | Follow up of missed workdays and caregivers | Costs and charges | USD | 2013 | 2 years cumulative gain 0.43 | 33,417 | 5,702 | 39,120 | 90,977 | - | - | - | - | - |
| Jazini et al. (2018) | SF-6D estimated from ODI | Hospital financial department | - | Costs | USD | 2018 | 2 years cumulative gain 0.14 | 31,882 | - | - | - | - | - | - | - | - |
| Tye et al. (2018) | EQ-5D | Hospital financial department | - | Charges | USD | 2014 | Mean gain 0.10 | 8,964 | - | - | - | - | - | - | - | - |
| Lyons et al. (2019) | - | Hospital financial department | - | Charges | USD | 2014 | - | - | - | - | - | - | 21,838 | - | - | - |
| Djurasovic et al. (2020) | EQ-5D | Hospital financial department | - | Costs | USD | 2020 | Mean gain 0.14 | 15,867 | - | - | - | - | - | - | - | - |
| Ver et al. (2020) | - | Hospital financial department | - | Costs | USD | 2020 | - | 18,204 | - | - | - | - | - | - | - | - |

Abbreviations: CPT codes = Current Procedural Terminology codes, DRG codes = Diagnosis Related Group codes, EQ-5D = EuroQol-five Dimensions, ODI = Oswestry Disability Index, PLIF = Posterior Lumbar Interbody Fusion, QALY = Quality Adjusted Life Years, SF-6D = Short Form-six Dimensions, TLIF = Transforaminal Lumbar Interbody Fusion, USD = United States Dollars.
