# Supplementary material for: Comparison of (Partial) economic evaluations of transforaminal lumbar interbody fusion (TLIF) versus Posterior lumbar interbody fusion (PLIF) in adults with lumbar spondylolisthesis: A systematic review
Source: PLoS One. 2021 Feb 11;16(2):e0245963. doi: 10.1371/journal.pone.0245963 (PMC7877595; doi:10.1371/journal.pone.0245963)
Supplement: S1 File — (DOCX) [file pone.0245963.s003.docx]

**S1 File. Search results.**

1. Pubmed:

- Search: ((lumbar spondylolisthesis) OR (lumbar instability) OR "Spondylolisthesis"[Mesh]) AND ((Transforaminal lumbar interbody fusion) OR TLIF OR (Posterior lumbar interbody fusion) OR PLIF OR "Spinal Fusion/methods"[Mesh]) AND (cost OR "Costs and Cost Analysis"[Mesh] OR "Cost-Benefit Analysis"[Mesh] OR (economic evaluation) OR pricing OR (cost-utility analysis) OR (cost-effectiveness analysis) OR cost-effectiveness)
- Filter: none

- Hits: 116

2. Embase:

- Search: (lumbar spondylolisthesis OR lumbar instability OR Spondylolisthesis) AND (TLIF OR transforaminal lumbar interbody fusion OR PLIF OR posterior lumbar interbody fusion OR Spinal Fusion) AND (cost OR Cost Analysis OR Cost-benefit analysis OR economic evaluation OR Pricing OR cost-utility analysis OR cost-effectiveness analysis OR cost-effectiveness).mp.

- Filter: none

- Hits: 179

3. Cochrane Library: http://www.cochrane.org/

- Search: Lumbar interbody fusion AND cost.

- Filter: none

- Hits: 58

4. Current Controlled Trials (CCT): http://controlled-trials.com/

- Search: Lumbar interbody fusion AND cost
- Filter: none

- Hits: 2

5. ClinicalTrials.gov: http://clinicaltrials.gov/

- Search: Lumbar interbody fusion AND cost
- Filter: none

- Hits: 7

6. NHS Centre for Reviews and Dissemination (CRD): http://www.york.ac.uk/inst/crd/

- Search: Lumbar interbody fusion AND cost
- Filter: none

- Hits: 13

7. Web of science: https://apps.webofknowledge.com/
- Search: ((lumbar spondylolisthesis) OR (lumbar instability) OR Spondylolisthesis) AND (TLIF OR (transforaminal lumbar interbody fusion) OR PLIF OR (posterior lumbar interbody fusion) OR (Spinal Fusion) OR (fusion methods)) AND (cost OR Cost-benefit OR (economic evaluation) OR Pricing OR cost-utility OR cost-effectiveness)

- Filter: none

- Hits: 313

8. Econlit:

- Search: ((lumbar spondylolisthesis) OR (lumbar instability) OR "Spondylolisthesis"[Mesh]) AND ((Transforaminal lumbar interbody fusion) OR TLIF OR (Posterior lumbar interbody fusion) OR PLIF OR "Spinal Fusion/methods"[Mesh]) AND ((cost) OR ("Costs and Cost Analysis"[Mesh]) OR ("Cost-Benefit Analysis"[Mesh]) OR (economic evaluation) OR pricing OR (cost-utility analysis) OR (cost-effectiveness analysis ) OR (cost-effectiveness))
- Filter: none
- Hits: 5
