# Supplementary material for: Comparison of (Partial) economic evaluations of transforaminal lumbar interbody fusion (TLIF) versus Posterior lumbar interbody fusion (PLIF) in adults with lumbar spondylolisthesis: A systematic review
Source: PLoS One. 2021 Feb 11;16(2):e0245963. doi: 10.1371/journal.pone.0245963 (PMC7877595; doi:10.1371/journal.pone.0245963)
Supplement: S2 File — (DOCX) [file pone.0245963.s004.docx]

**S2 File: Risk of bias and methodological quality of economical evaluations (CHEC-list)**

Table 1. Risk of bias assessed with the bias assessment tool of the Cochrane Handbook for Systematic Reviews of Interventions.

| **Author** | **Design** | **Date collection** | **Selection bias** | **Performance bias** | **Detection bias** | **Attrition bias** | **Reporting bias** | **Total score** |
| --- | --- | --- | --- | --- | --- | --- | --- | --- |
| Whitecloud et al. (2001) | Cost analysis | Retrospective | + | + | + | +/- | +/- | high |
| Wang et al. (2010) | Cost analysis | Retrospective | + | + | + | +/- | - | high |
| Adogwa et al. (2011) | Cost-effectiveness study | Prospective | + | + | + | +/- | - | high |
| Lucio et al. (2012) | Cost analysis | Retrospective | + | + | + | +/- | - | high |
| Parker et al. (2012) | Cost-effectiveness study | Prospective | + | + | + | - | - | high |
| Sulaiman et al. (2014) | Cost analysis | Prospective | + | + | + | +/- | +/- | high |
| Singh et al. (2013) | Cost analysis | Retrospective | + | + | + | +/- | - | high |
| Parker et al. (2014) | Cost-effectiveness study | Prospective | + | + | + | + | - | high |
| Christensen et al. (2013) | Cost-effectiveness study | Prospective | - | + | + | - | - | low |
| Gandhoke et al. (2015) | Cost-effectiveness study | Prospective | + | + | + | - | +/- | high |
| Kim et al. (2017) | Cost-effectiveness study | Prospective | + | + | + | + | - | high |
| Jazini et al. (2018) | Cost-effectiveness study | Retrospective | + | + | + | - | - | high |
| Tye et al. (2017) | Cost-effectiveness study | Retrospective | + | + | + | - | - | high |
| Lyons et al. (2019) | Cost analysis | Retrospective | + | + | + | - | - | high |
| Djurasovic et al. (2020) | Cost-effectiveness study | Retrospective | + | + | + | +/- | +/- | high |
| Ver et al. (2020) | Cost analysis | Retrospective | + | + | + | +/- | +/- | high |

Table 2. Methodological quality of economical evaluations assessed with the Consensus Health Economic Criteria (CHEC) list.

| **Article** | **1. Describes study population** | **2. Competing alternatives** | **3. Research question** | **4. Economic study design** | **5. Time horizon** | **6. Perspective** | **7. Relevant costs** | **8. Appropriately measured costs** | **9. Appropriately valued costs** | **10. Relevant outcomes** | **11.Appropriately measured outcomes** | **12.Aappropriately valued outcome** | **13. ICER** | **14. Discounted** | **15. Sensitivity analyses** | **16. Correct conclusions** | **17. Generalization** | **18. Conflict of interest** | **19. Ethical issues** | **Total +** |
| --- | --- | --- | --- | --- | --- | --- | --- | --- | --- | --- | --- | --- | --- | --- | --- | --- | --- | --- | --- | --- |
| Whitecloud et al. (2001) | + | + | + | - | +/- | - | - | +/- | - | +/- | + | - | - | - | - | - | - | - | - | 5.5 |
| Wang et al. (2010) | + | + | +/- | - | +/- | - | - | +/- | - | - | + | - | - | - | - | + | + | +/- | - | 7.0 |
| Adogwa et al. (2011) | + | - | + | - | + | + | + | + | +/- | + | + | + | - | - | - | + | - | + | - | 11.5 |
| Lucio et al. (2012) | + | - | + | - | + | - | +/- | +/- | +/- | + | - | - | - | + | - | - | - | +/- | - | 7.0 |
| Parker et al. (2012) | + | + | + | + | + | + | + | + | +/- | + | + | + | + | - | - | + | - | + | - | 14.5 |
| Sulaiman et al. (2014) | + | + | + | +/- | + | - | +/- | +/- | +/- | - | + | + | - | - | - | + | - | + | - | 10.0 |
| Singh et al. (2014) | + | + | + | - | + | - | + | +/- | - | + | + | + | - | - | - | + | + | + | - | 11.5 |
| Parker et al. (2014) | + | + | + | + | + | + | + | + | +/- | + | + | + | + | - | - | + | - | + | - | 14.5 |
| Christensen et al. (2014) | + | - | + | + | + | + | +/- | +/- | + | + | +/- | + | + | +/- | + | + | + | + | - | 15.0 |
| Gandhoke te al. (2015) | +/- | - | + | + | + | + | +/- | + | - | + | + | + | + | - | +/- | + | + | + | - | 13.5 |
| Kim et al. (2017) | + | +/- | + | + | + | + | + | + | - | + | + | + | - | - | - | +/- | - | + | - | 12.0 |
| Jazini et al. (2018) | + | + | + | + | + | - | +/- | - | - | + | +/- | + | + | - | - | + | - | +/- | - | 10.5 |
| Tye et al. (2018) | + | - | +/- | + | + | - | +/- | +/- | - | + | + | + | - | + | - | +/- | - | +/- | - | 9.5 |
| Lyons et al. (2019) | + | +/- | - | - | + | - | +/- | + | - | + | + | - | - | - | - | - | + | + | + | 9.0 |
| Djurasovic et al. (2020) | + | +/- | +/- | + | + | - | + | +/- | - | + | + | + | - | - | - | +/- | - | +/- | - | 9.5 |
| Ver et al. (2020) | + | - | + | - | + | - | - | +/- | - | + | + | - | - | - | - | + | - | +/- | - | 7.0 |
